# Area and Resource Utilization of Group-Housed Horses in an Active Stable

**DOI:** 10.3390/ani11102777

**Published:** 2021-09-23

**Authors:** Frederik Hildebrandt, Kathrin Büttner, Jennifer Salau, Joachim Krieter, Irena Czycholl

**Affiliations:** 1 Institute of Animal Breeding and Husbandry, Kiel University, 24118 Kiel, Germany; kathrin.buettner@vetmed.uni-giessen.de (K.B.); jsalau@tierzucht.uni-kiel.de (J.S.); jkrieter@tierzucht.uni-kiel.de (J.K.); iczycholl@tierzucht.uni-kiel.de (I.C.); 2 Unit for Biomathematics and Data Processing, Faculty of Veterinary Medicine, Justus Liebig University, 35392 Giessen, Germany

**Keywords:** horse, group housing, active stable systems, area utilization, GPS

## Abstract

**Simple Summary:**

Horses are increasingly being kept in group stables where they are given the opportunity to move freely and to contact conspecifics. This study examined how the horses of a large group move in an active stable and which areas or resources they use over a period of 7.5 months with the help of linear mixed models. Therefore, a grid with the size of 3 × 3 m per square was applied. The horses used, on average, 53.2 ± 19 different squares per hour. It was shown that observation day (*p* < 0.001) and covariate age (*p* < 0.001) had significant effects on squares visited per hour. Sex (*p* = 0.30) and breed (*p* = 0.65) had no significant effects. A large variation in the squares visited per hour was determined for the individual horses. Most frequently visited were the individual resources, such as the feed stalls or lying halls. An open tarp skin lying hall was preferred over a metal hall. The shelters were only partly popular. An overview of the usage frequency of the paddock could be created by using heatmaps. Overall, specific conclusions about the use of individual areas and potential for improvement could be pointed out.

**Abstract:**

The aim of this study was to analyze the utilization of different stable areas of a total of 52 group-housed horses as well as their preferred stable parts and the use of resources. The study was situated in a “HIT Active Stable^®^” in Northern Germany for a period of 227 observation days. After dividing the whole farm area in a grid of 3 × 3 m, the dataset was examined with and without the pasture area. Furthermore, linear mixed models were applied. On average, horses used 53.2 ± 19 different squares per hour. The observation day (*p* < 0.001) and the covariate age (*p* < 0.001) had significant effects on the different squares visited per hour. No significant effects were found for sex (*p* = 0.30) and breed (*p* = 0.65) as only geldings and no stallions were part of the group and the distribution of the breeds was unfavorable. The random effect animal showed that the horse-individual estimates from −19.2 to 17.6 different squares visited per hour were quite large. Furthermore, it could be shown that the horses used resources such as feed stalls with a frequency of up to 0.14% more than other paddock areas without resources. Open lying halls with tarp skin were also preferred over the metal hall. The shelters were only partly popular. Use could be visualized with the help of heat maps. This study gives a good overview of the use of individual areas and resources and possible improvements.

## 1. Introduction

In Germany, keeping horses in groups is becoming an increasingly common practice. This type of husbandry enables these highly social animals to better live out their species-specific behaviors, such as all-day movement and the possibility of social interaction with conspecifics. The positive effect of group housing and social interactions is adequately described in the literature [1,2,3,4,5,6]. With the help of this housing system, stereotypes and abnormal behavior [4], as well as stress, can be reduced and so have a positive effect on the well-being of the horses housed in it [7]. Furthermore, Szivacz [8] reported an enhancement of respiratory and gastrointestinal diseases as well as orthopedic complaints by changing husbandry systems from single boxing to group housing. Christensen et al. [5] found out that two-year-old Danish warmblood breeding stallions in group housing showed less aggressiveness but more social grooming and play behavior compared to single housing by inclusion in a group over a period of six weeks. Moreover, the lack of contacts with conspecifics can lead to higher difficulty of horse handling [6]. Furthermore, an increased frequency of undesirable behavior towards humans was observed, as well as a negative effect on the learning ability of horses. [9,10].

It is also important to consider possible risks and disadvantages of group housing because in contrast to single housing, horses in group housing systems are not always immediately available for the owners. Some horse owners stated that there is an increased risk of injury due to increased gamboling and agonistic behavior in groups [11,12]. Important factors with regard to conflicts and injuries are the available area per animal [13] and the group composition. Keeling et al. [14] determined that the injury level is less influenced by sex and age and more by breed. Furthermore, the authors identified that Icelandic horses had fewer injuries than warm-blooded horses after mixing groups. Rank fights are also of particular importance in pension stables, where a certain instability due to newcomers and disposals is unavoidable. Here, the literature is contradictory: on the one hand, there are reports of positive effects and, on the other hand, of injuries caused by rank fights. To minimize potential risks, stables should be optimally managed to avoid negative interactions [15], and constructed to give horses escape options to move away and avoid dead ends, bottlenecks and corners [11,16]. Furthermore, a stable can be optimized by installing functional areas designed to create additional incentives for movement [11,16,17]. This concept is implemented throughout HIT active stables^®^ (Hinrichs Innovation + Technik, Weddingstedt, Germany) [18]. Increased movement is the basis of such stables and was analyzed in a previous study [19]. Thus far, hardly any research has been conducted on whether the horses actually use all functional areas as intended by the manufacturers. The aim of this study was to analyze whether horses use all stable areas and by which factors this is influenced. Here, the subdivision of the stable into squares offers a useful evaluation possibility. Furthermore, the question arises regarding which areas are most frequently visited and whether there are areas within the stable that are rarely used. Here, it is necessary to examine the use of important functional areas and resources. Overall, this study contributes to knowledge concerning optimized management in group housing systems of horses with special movement concepts.

## 2. Materials and Methods

### 2.1. Horses, Surroundings and Observation Period

**Figure 1:**
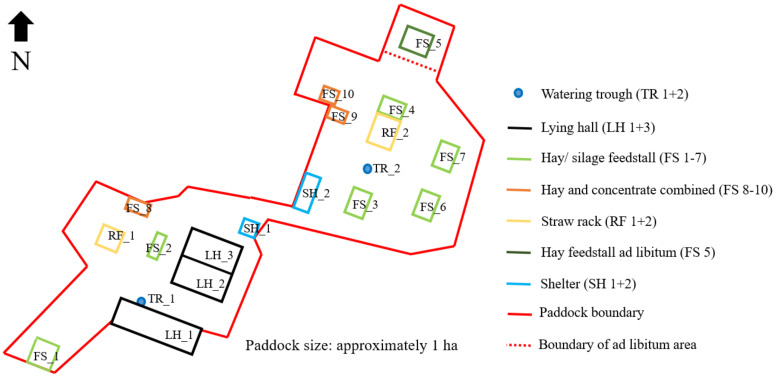
Schematic diagram of paddock area and resources.

In a “HIT Active Stable^®^” [18] in Northern Germany, privately owned mares and geldings of various breeds (e.g., German Warmblood, German Riding Pony, Arabs and German Trotter, special breeds and mixed) were held as one group. Detailed information about the horses analyzed is given in the Supplementary Materials (Table S1). In total, the herd consisted of 50 horses in the beginning, of which 40 horses initially participated in the study. In the further progress of the study, 12 newcomer horses were integrated into the herd, all of which participated in the study. The ages of all participants differed between 2 and 29 years throughout the whole group. Ten additional horses were in the group the whole time but were not included in the study since the owners refused their participation in the study for various reasons. Horses could move freely throughout the whole day. In the end of the investigation, the paddock area had a size of approximately one hectare, i.e., about 160 m^2^ paddock area for each horse (Figure 1). Access to hay and concentrated feed in different feed stalls according to individual needs was computer controlled by subcutaneous computer chips or sensors on nylon safety collars. Moreover, free admission to straw in racks was available to all horses. Three lying halls bordered in black in Figure 1 were located in the paddock (2 × 160 m^2^ made of tarp skin, 1 × 250 m^2^ made of metal). During winter, the bedding of the metal hall was changed to a substrate of sunflower cobs. Two drinking troughs in the paddock set up as double drinking troughs allowed, theoretically, two horses per drinking place to drink at the same time. In the summertime, the horses had pasture access twice a day (in the morning and evening) for about 90 min each. The pasture areas were directly adjacent to the paddock and were changed according to the stock of grass.

In total, a period of 227 days was under observation including 159 days in summer (June–November) and 68 days in winter (November–middle of January). During the observation time, the paddock was redesigned. An ad libitum area (FS_5) with hay for individual horses was created in the very north of the paddock. Two roughage feed stalls (FS_6+7) were also built in the northeastern area of the paddock.

### 2.2. Data Loggers, Attachment and Observation Period

**Figure 2:**
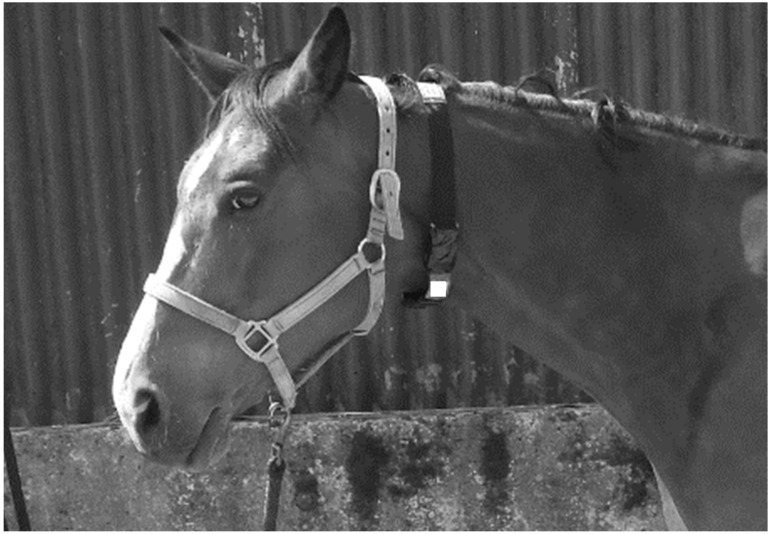
Bay horse with safety collar and attached data logger.

All studied horses were equipped with data loggers BT-Q1000XT from QSTARZ^®^ (Taipei, Taiwan), covered with duct tape on nylon safety collars as evident in Figure 2. A randomly ascending number was assigned to each horse to keep the data anonymous.

The logger collars were attached in a way that they so could be worn 24 h a day and did not disturb during the daily routine, such as moving, eating, drinking and playing. Outside the paddock, the collars were taken off during times of working or strolling with their owners. As a result, only the time on paddock and pasture was considered in this study. We chose 0.1 Hz (a recording point every 10 s) as the sampling frequency of the GPS data loggers. The data contained local and UTC date and time, latitude, longitude, height, as well as speed and distance. Due to battery performance, the loggers were changed every 36 h.

### 2.3. Data Filtering and Statistical Analysis

#### 2.3.1. Data Filtering

Initially, GPS measurements located outside of the most northern, eastern, western and southern position coordinates of the farm were extinguished. Data from heavily demolished or incorrectly adjusted loggers were also erased. According to the horses’ physical abilities, speeds of more than 50 km/h and distances of >140 m per 10 s were filtered out. Furthermore, a file with the individual positions for each day and horse was created. A grid of squares with a size of 3 × 3 m for the whole paddock and pasture area was generated and all recordings outside the paddock and pasture borders were deleted. As a result, the position of each horse in a 3 × 3 m square was determined every 10 s. Further filtering details can be found in Salau et al. [20]. In other previous investigations of horses’ contacts, an Euclidean distance of 3 m between horses (based on data loggers attached to collars on horses’ necks) had been deemed the most useful spatial contact definition [21]. This definition also served as a basis for the choice of the grid size.

#### 2.3.2. Visited Squares per Hour

In order to investigate the usage of different farm area parts, the number of squares visited per hour for each horse and day were calculated with a linear mixed model by using SAS^®^ 9.4 software (Cary, NC, USA) [22]. Horses’ usage of different paddock parts was investigated in two ways. Once with the involvement of the pastures and once without their involvement. Consequently, observation days with less than 12 h of data recording (compliant with 4320 observations per day) were excluded from the investigation of the different squares used per hour. After data filtering considering the dataset with pasture, 8011 days of approx. 10,147 expected days were left. Since without pasture, often no 12 h of daily data were collected, the dataset without pasture was reduced to 5101 days. In order to develop this model, fixed effects were added to the model in a stepwise manner. Therefore, the Akaike’s information criteria corrected (AICC) in combination with the Bayesian information criteria (BIC) were used to compare the different models. The ones with the smallest AICC and BIC values were chosen. Finally, the following model was developed:

$$y_{ijkl}=µ+B_{i}+S_{j}+D_{k}+A_{l}\left(B_{i}\right)+b\;age_{ijkl}+e_{ijkl},$$

where y_ijkl_ was the squares visited per hour and µ is the overall mean. The fixed effect B_i_ described the fixed effect of the ith breed (i = warmblood, pony, other). Breeds with a low number of individuals (including Arabs, German Trotter, special breed and mix) were grouped into one category, labeled ‘other’. The classification of the individual class of breeds was as follows: 31 warmblood horses, 6 ponies and 15 other (including Arabs and German Trotter, special breeds and mixed horses. The division into these three groups was based on the rather unfavorable distribution of the breeds. Furthermore, S_j_ characterized the fixed effect of the jth sex (j = gelding, mare) as well as D_k_ as the fixed effect of the kth observation day (k = 1, …, 227). Due to the repeated observations, animal A_l_, which described the lth animal (l = 1, …, 52) nested by the ith breed (i = warmblood, pony, other) was included in the model as a random effect. In order to take age into account, the covariate age was involved in the model and e_ijkl_ presented the residual term. The significance of differences between the Least Square Means (LSM) was adjusted with the help of the Bonferroni-correction and the significance level was *p* < 0.05. The residuals were graphically investigated and approximately normally distributed.

#### 2.3.3. Preferred Farm Parts and Heatmaps

In a second step, the parts of the farm area preferred by the horses were investigated. For this purpose, the individual resources, such as lying halls, feed stalls, etc., were assigned to individual squares according to their location. As above, the dataset was examined with and without pasture in each case. The residuals were analyzed graphically and deviations in the values especially at the edges were found. Due to the rather high kurtosis, there was a deviation from the normal distribution. This was to be expected as the pasture had limited access and took up a very large part of the squares. As soon as the pasture data was excluded from the dataset, the kurtosis was reduced by a factor of four. We refrained from transforming the values, as the distribution without pasture was clearly better and thus a uniform presentation of results could proceed. Linear mixed models (PROC MIXED) were developed in the same way as in the first part:

$$y_{ijk}=µ+D_{i}+L_{j}+e_{ijk},$$

the variable *y_ijk_* described the frequency of usage of the individual square per day and µ presented the overall mean. Two fixed effects were included in the model. D*_i_* is the fixed effect of the *i*th observation day (*i* = 1, …, 227) and *L_j_* characterized the location of the square on the farm (*j* = feed stall No. 1–7, concentrate feed stall 1–3, lying halls 1–3, straw rack 1–2, shelter 1–2, trough 1–2, remaining paddock area and the pasture) evident in Figure 1. Due to the additional pasture area, the horses used 34,381 different squares with and in contrast to 3107 different squares without pasture. In the further course, heat maps of the paddock in summer and winter were created with PROC SGPLOT by using SAS^®^ 9.4 software to identify visually the high frequented areas of the HIT active stable^®^ investigated.

### 2.4. Ethical Statement

The horses included in the study were privately owned and normally kept in a pension stable. During the observation period, the horses were not restricted in their normal behavior and the horses were able to carry out their daily routine. The animals were not injured or in pain during the study period. The authors ensured that the “German Animal Welfare Act” (German designation: Tierschutzgesetz [TierSchG]) and the “German Order for the Protection of Animals used for Experimental Purposes and other Scientific Purposes” (German designation: Tierschutz-Versuchstierverordnung [TierSchVersV]) were applied. The owners of the individual horses had previously agreed to the participation of their animals.

## 3. Results

### 3.1. Visited Squares per Hour

Including data with pasture, each horse was located in 53.2 ± 19 squares/h on average. No significant effect was found for the breed (*p* = 0.65) (Table 1). Warmblood horses visited 53.1, ponies 54.9 and other horses 54.8 different squares/h. In addition, geldings (55.3 squares/h) and mares (53.3 squares/h) visited nearly the same number of squares (*p* = 0.30). When considering age, it was found that with each increased year of life, the squares per hour visited decreased by 0.6 (*p* < 0.001). Similar significances, but with lower Least Square Means (LSM), were also found for the dataset without pasture.

**Figure 3:**
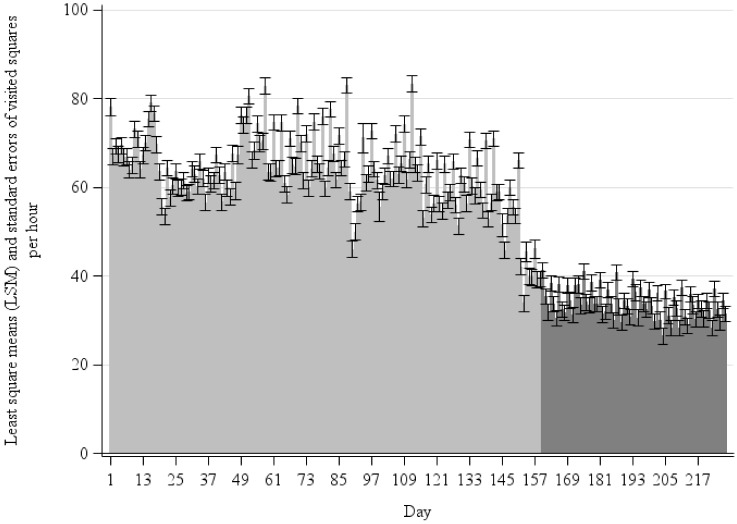
Least square means (LSM) and the standard errors of the squares visited per hour depending on the individual observation day including the pasture (light gray: summer/dark gray: winter).

However, a significant effect of the LSM could be determined for the observation day (*p* < 0.001) (Figure 3). During summer with pasture access (light gray), the estimates of squares visited per hour ranged from 33.8 to 83.4. The observed values in winter (dark gray) were smaller. They ranged between 26.5 and 41.1 squares/h. Generally, a high daily variation could be determined, especially in summertime. When considering only the paddock area, similar significant results with lower LSMs in summer were obtained (not illustrated).

**Figure 4:**
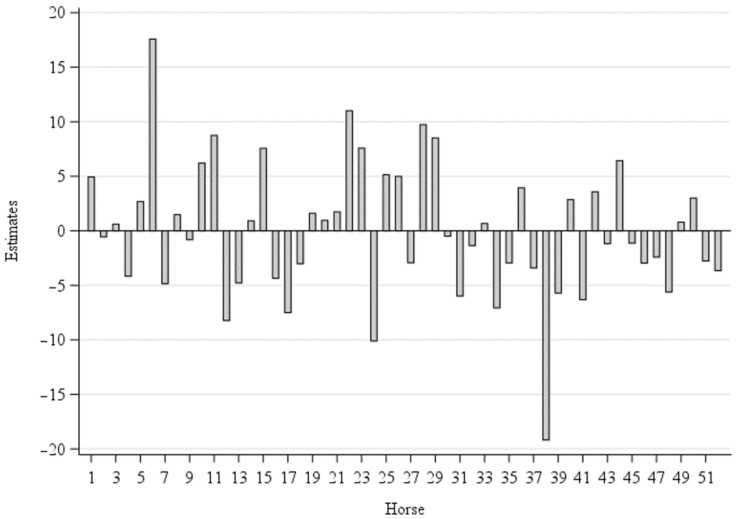
Estimates (Squares visited per hour) of the random effect animal.

Figure 4 presents the animals’ individual differences of visited squares. Due to their later inclusion in the group, only data for the winter could be included from horse 40 onwards. The differences for the individual animals presented ranged between −19.2 (Horse 38) and 17.6 (Horse No. 6) squares per hour.

### 3.2. Preferred Farm Parts and Heatmaps

**Figure 5:**
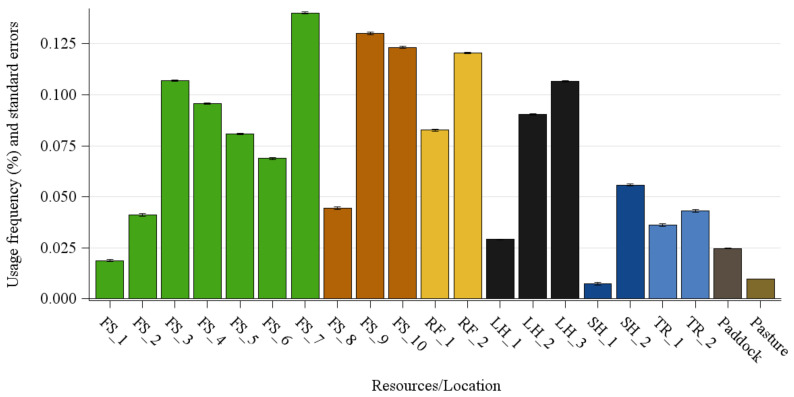
Usage frequency (%) and standard errors of different locations/resources of the farm divided into feed stalls (FS/green, orange), straw racks (RF/yellow), lying halls (LH/black), shelters (SH/dark blue), watering troughs (TR/light blue), paddock (dark brown) and pasture (light brown).

The evaluation of the second model revealed significant differences in the LSMs of day (*p* < 0.001) and location (*p* < 0.001) in both variants with and without consideration of the pasture. Due to the very high number of squares, the percentage values are very low. The LSMs of the individual days were between 0.06 and 0.08%. The usage frequency of the different resources of the farm is shown in Figure 5. The use of the individual feeding stalls is quite different. Feed stall 1 (FS_1) was only available for a few months in summer. It can be seen that feed stall 7 (FS_7) was used most frequently with a frequency of 0.14%. In general, both straw racks (RF_1+2) were used quite frequently. With regard to the lying halls, it can be seen that halls No. 2 and 3 (made of tarp skin) were predominantly used (0.09%, respectively, 0.11%) compared to lying hall No. 1, which is made of metal. Shelter 1 with a frequency of 0.01% was rarely used. In addition, the horses spent about the same amount of time at both drinking troughs with a tendency towards more use of watering trough No. 2. In general, the paddock area (without resources) was used with a frequency of 0.2%. The grazing area, which was accessible for a limited time and only in summer, was visited with a frequency of 0.01% and took up the most squares by far. For visualization purposes, the results are shown in the following figures, divided into summer and winter.

**Figure 6:**
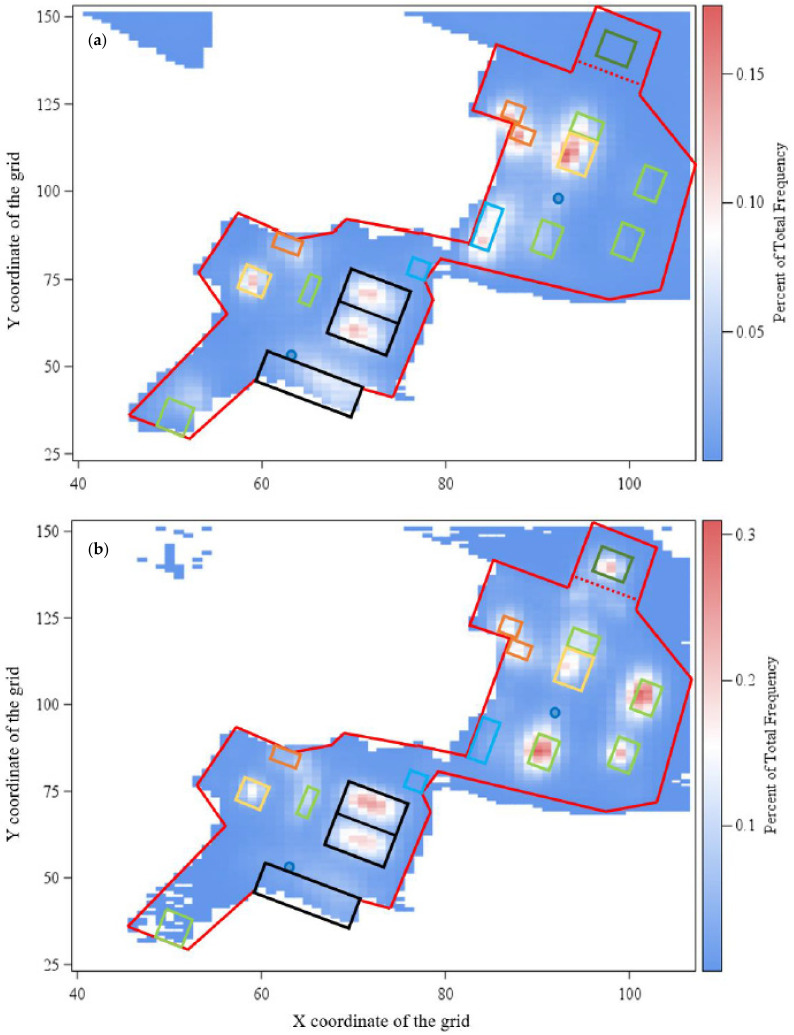
Heatmaps and scale of paddock area in summer (**a**) and winter (**b**).

Figure 6a presents the usage of the paddock in summer with the help of a heatmap. Generally, the individual squares were visited with a frequency of, at most, approximately 0.3%. It can be clarified again that the animals spent most of their time along the resources. Although the whole paddock was used, some areas were used to a lesser extent. In particular, the two straw racks outlined in yellow were used highly frequently, as were lying halls 1+2 outlined in black and the area between them. The ad libitum area (FS_5) and hay feed stalls on the right (FS_6+7), outlined in light green, were under reconstruction during data collection and were therefore less frequented than the permanently, freely accessible areas. Furthermore, the squares near the troughs were not used to a high degree. The paddock usage in winter is demonstrated in Figure 6b. The straw racks (yellow lined) were also highly frequently visited. The new feed stalls (light green/FS_6+7) had a high gain in usage compared to summer, as did the older ones. In addition, the newly established ad libitum area (FS_5) was highly frequented by the horses assigned to it. This area did not yet exist in the summer. Furthermore, the shelters were not widely used as a place to stay. The squares used in the pasture in winter result from individual walks of the horse owners with their horses.

## 4. Discussion

### 4.1. Logger Performance, Experimental Method and Limitations

The farm area could be divided and the whereabouts of horses could be determined expediently with the help of the chosen grid of 3 × 3 m. However, the determination of the location at the resources should be considered carefully since the choice of 3 m as the square size could also detect the areas immediately adjacent to the resources. Additionally, it must be taken into account that loggers also have a certain inaccuracy, which can be amplified by roofs or other disturbances. Moreover, owners who walked their horses on the farm area outside of the paddock area without removing the collars were problematic for the data evaluation. This occurred especially in winter, when a trail just outside the paddock area was frequently used for short walks. For this reason, a few data points were located outside the paddock. These can be seen in Figure 6b in the upper left corner. However, a large dataset was obtained despite single losses and destruction of the loggers by the horses. Geldings especially often destroyed or lost their loggers. Nevertheless, their number was relatively small. Furthermore, it could not be recorded whether the horses had lost their collars and these were lying in the paddock and were then attached to the horses again by the owners themselves. Moreover, some owners worked with their horses outside the paddock without taking off the collars, which could also have had an influence on the data due to tracks just at the border. In winter, low temperatures shortened the longevity of the batteries in the loggers.

### 4.2. Visited Squares per Hour

The fixed factors sex (*p* = 0.30) and breed (*p* = 0.65) had no significant effect on different visited squares/h. This confirmed previous results in Hildebrandt et al. [19] with regard to walking distances. To prevent uncontrolled reproduction and to avoid increased aggression due to sexually motivated behavior, only mares and geldings were kept within the group. Dominance relationships between stallions are contested more often than between mares [23]. However, due to the mainly destroyed transmitters of priority geldings, one could expect that their play behavior would be higher and rougher, but this was not represented by increased values for squares/h. The assumption is that the horses interacted rather inertly and did not move larger distances while doing so. Nevertheless, it is rather unproblematic to keep both sexes in one group [24]. The lack of differences in terms of breed was certainly due to the chosen classification. The great diversity of breeds, especially in the classification “others”, probably did not lead to significant results. The group “others” included 15 horses of various breeds, such as Trotters, Arabs or diverse special breeds, such as Frisian, Paint Horses and American Quarter Horses, which are breed-typically very different. The classification "warmblood" was the largest with 31 individuals. The second classification contained six ponies. One possible interpretation is that all horses had to cover the same distances between the resources, so that the individual effect should be small.

The factor observation day had a significant effect on the squares visited per hour. Certainly, the possibility of visiting the pasture has a high influence. This can be seen especially by considering the summer data. Activity phases are longer due to greater daylight periods. While grazing, horses typically move physiologically forward bit by bit. The occasional escape from insects could also have influenced the number of visited squares. Considering only the paddock without pasture, the observation day also had a significant effect. Furthermore, the inclusion of new horses and the establishment of a new rank order also had an effect, which had already been proven in Hildebrandt et al. [19]. Individual daily circumstances such as the surrounding conditions and especially the interaction with the owner presumably influenced the daily results. The factor age had a significant effect. Per increasing year of age, the horses visited 0.6 fewer squares per hour (*p* < 0.001). At a younger age, horses have an increased urge to play and explore, which leads to more movement within the barn. This corresponds with Krueger et al. [25], who reported that horses show less exploration behavior with higher age. Furthermore, older horses are more susceptible to illness, such as arthrosis, which could decrease the joy of movement. Another reason could be that older horses drop in rank over time and no longer visit as many areas, as these could be blocked by higher-ranking horses.

When considering the random effect animal in Figure 4, it can be seen that the use of the squares shows quite high animal-individual differences. Horse ’38’ (warmblood) had the lowest number of squares/h. This horse was the only horse that practiced the abnormal behavior known as crib biting. Abnormal behavior is more likely to be seen in horses kept singly with very small contact to conspecifics [26]. However, this behavior is often residual-reactive and this horse came to the farm with this stereotype. The only Norwegian horse in the group (No. 24) also sought out few squares. It was also the horse that covered the least distance per day [19]. On the other hand, a Trotter gelding (classified as other) (No. 6) visited the most different areas, followed by a warmblood gelding (No. 22).

### 4.3. Preferred Farm Parts and Heatmaps

In general, the frequencies of the visited squares in Figure 6a,b are relatively low with values below 0.3%. This is due to the number of potentially different squares being quite high with a value of 34,381 considering the pasture area. Nevertheless, the horses used all areas of the pasture and paddock. Since the pasture areas were only changed when the grass was completely grazed down, the horses had to visit all sections of the pastures. In addition, the horses generally had a high urge to move, which could be better satisfied by the open area. A strong increase in the frequency of visits was observed along the resources. Figure 5 showed that the lying halls strewn with straw (LH 2+3) were used highly frequently. The southern lying hall was less frequently used. This hall was a former cattle barn made of metal, which had smaller entrances and supports in the middle. The air circulation was rather poor and the room dark. Therefore, it is advisable to build new large lying halls made of tarp skin, as they are better used by the horses. By nature, horses have several short rest periods that are spread out in intervals throughout the whole day. Thereby, they rest in standing or lying positions for 5–9 h a day [27]. Additionally, the lying halls could be used for protection from the weather or partly from conspecifics. Another reason for the high frequency of usage could be that the horses did not only use the lying halls as a resting area but used the straw bedding as roughage feed. Although, in general, it has proven positive when horses are given the opportunity to eat roughage all day long, such as straw bedding [28], this might be questionable with regard to the disturbance of resting animals. From these data, the behavior of the animals performed in the lying halls (e.g., resting behavior, roughage intake) could be analyzed, but it should be analyzed in more detail in further studies. The group size is also important. The study of Rose-Meierhöfer et al. [29] on young horses found out that particularly large groups of 23 horses showed higher lying times compared to a group size of 11 animals. In this study, the group size was even larger, hence a high lying time could be expected, which could again explain the high usage of the lying halls. The first shelter was seldom used as it was very small, only had a small roof, and offered little protection. Shelter No. 2 (SH_2) was also used for protection from the sun during hot temperatures, especially in summer. At higher temperatures, the animals sought shelter No. 2 more often, because it was cooler in the shelter than outside [30]. In winter, however, the utilization of the shelters was rather low in this study. Snoeks et al. [30] also investigated horses’ behavior in a temperate climate and reported increased shelter use in cold temperatures, which was not observed in this study. Presumably, they tended to use the lying halls, since these were also protected from the wind on the sides. Maybe their horse blankets or the protection in the lying halls were a sufficient protection for the horses.

Horses are adapted to temperate grasslands. In the natural environment they spend more than 14 h of a day foraging [31]. The importance of foraging is also reflected in the high use of feed stalls and straw racks. In this study, the whole group could not eat synchronously, which is rather negative because horses would eat simultaneously in nature [32]. Nevertheless, several horses were able to eat at the same time in the feed stalls. The horses had individual access to the feed stalls, controlled by computer chips according to their body condition and the owner’s default. Some horses gathered around in the immediate surroundings of the feed stalls trying to pick up additional feed. Thereby, there might be an increased risk of aggressive interactions due to a gathering of horses in the waiting area [33]. Additionally, an ad libitum hay area (FS_5) was established during the monitoring period for older horses or less conditioned horses, so that they could take hay without limitation and disturbances according to their needs. The area was partially barred for grazing and construction during the summer. The feed stall (FS_1) and the small area in the southwestern part of the paddock was closed at the beginning of winter. Therefore, the frequency in Figure 5 was very low. Furthermore, the straw racks were often visited on the paddock throughout the whole year. There, the animals had the opportunity to take in raw fiber without restriction and it served as protection from the wind. The consumption of straw also serves as an occupation or stress relief, as well as to compensate for deficiencies in the roughage supply [34]. The use of the troughs was difficult to detect. In the summer, it can be seen that the drinking trough in the northeastern part of the paddock (TR_2) was visited quite frequently. This occurred mainly after grazing because of its location next to the pasture access.

The results showed that the horses use some areas higher frequently than others, especially the resources. The individual frequencies were only so low because of the high number in total. For example, the frequency of 0.14% of FS_7 resulted from the average of the assigned squares of the feed stall. Nevertheless, it can be seen that at least small differences along the individual areas could be identified. Thus, for example, potential for improvement could be determined, since the metal lying hall was used less frequently than the one with tarp skin. The metal hall had only small entrances and the horses could not observe their environment. The fact that all areas were used shows that the stable itself was generally well designed. Furthermore, it must be pointed out that these results are only valid for this specific active stable and that, for example, differences in the available space or a modification of the number of horses could be investigated in further studies. Likewise, in general, other stables which are structured or designed differently could be used. Special focus could also be made on group size, breeds sex. The results of the occupancy of the areas showed that the correct positioning of the individual resources is of great importance. This should be taken into account when planning the stable. Furthermore, the effect of the individual horses in the group should not be underestimated and stable areas should be individually adapted to the individual group.

## 5. Conclusions

The present study demonstrated the frequency of visits of the different squares and its influences. In general, it was quite variable as to how many different squares/h an animal visited. The individual horse, beside the day and the age, represented the biggest influence factor. Different events can influence movement in the stable. The utilization of farm areas was analyzed with the help of the second model and heatmaps. It can be speculated that by prioritizing the resources, the horses are motivated to move to reach necessary resources. Thus, it is important to separate the resources as far as possible from each other spatially to create an additional movement stimulation. In general, all paddock areas were used and, with a few individual exceptions, all horses basically used all areas.

## Figures and Tables

**Table 1:** Least Square Means (LSM), standard errors and *p*-values of the models divided in datasets with and without pasture.

	With Pasture			Paddock Only	
		**LSM and Standard Error**	***p*-Value**	**LSM and Standard Error**	***p*-Value**
Sex	Geldings	55.3 ± 1.4	0.30	39.7 ± 1.3	0.09
	Mares	53.3 ± 1.6		36.6 ± 1.6	
Breed	Warmblood	53.1 ± 1.2	0.65	37.1 ± 1.1	0.58
	Ponies	54.9 ± 2.7		38.2 ± 2.6	
	Other	54.8 ± 1.7		39.1 ± 1.7	
		**b-Value**	***p*-Value**	**b-Value**	***p*-Value**
Age		−0.6 ± 0.1	<0.001	−0.4 ± 0.1	<0.01

## Supplementary Materials

The following are available online at https://www.mdpi.com/article/10.3390/ani11102777/s1, Table S1: Characteristics of the horses.
