# Supplementary material for: Area and Resource Utilization of Group-Housed Horses in an Active Stable
Source: Animals (Basel). 2021 Sep 23;11(10):2777. doi: 10.3390/ani11102777 (PMC8532945; doi:10.3390/ani11102777)
Supplement: Supplementary file 1 [file animals-11-02777-s001.zip › animals-1349137-supplementary.pdf]

Supplementary Materials

Area and Resource Utilization of Group-Housed Horses in an Active Stable

Frederik Hildebrandt [1,*], Kathrin Büttner [1,2], Jennifer Salau [1], Joachim Krieter [1] and Irena Czycholl [1]

[1] Institute of Animal Breeding and Husbandry, Kiel University, 24118, Kiel, Germany; kathrin.buettner@vetmed.uni-giessen.de (K.B.); jsalau@tierzucht.uni-kiel.de (J.S.); jkrieter@tierzucht.uni-kiel.de (J.K.); iczycholl@tierzucht.uni-kiel.de (I.C.)

[2] Unit for Biomathematics and Data Processing, Faculty of Veterinary Medicine, Justus Liebig University, 35392, Giessen, Germany

* Correspondence: fhildebrandt@tierzucht.uni-kiel.de; Tel.: +49-431-880-1493

Table S1. Characteristics of the horses.

Horse No.	Age	Sex	Breed	Classification
1	19	w	Oldenburger	Warmblood
2	8	w	Hannoverian horse	Warmblood
3	16	m	German Riding Pony	Pony
4	22	w	Oldenburger	Warmblood
5	20	m	Arabian	Other
6	14	m	Trotter	Other
7	14	m	Arabian	Other
8	7	m	Hannoverian horse	Warmblood
9	24	w	German Riding Pony	Pony
10	20	w	Trotter	Other
11	10	w	Arabian	Other
12	24	m	Paint Horse	Other
13	14	m	American Quarter Horse	Other
14	18	w	Hannoverian horse	Warmblood
15	5	m	Hannoverian horse	Warmblood
16	20	m	Andalusian horse	Other
17	21	w	Warmblood	Warmblood
18	16	m	Holsteiner	Warmblood
19	29	w	Hannoverian horse	Warmblood
20	13	m	Mecklenburger	Warmblood
21	23	m	Cold-blooded horse x Arabian	Other
22	10	m	Trakehner	Warmblood
23	15	w	Oldenburger	Warmblood
24	11	w	Fjord	Pony
25	8	m	Oldenburger	Warmblood
26	18	m	German Riding Pony	Pony
27	14	m	Brandenburger	Warmblood
28	12	w	Rhinelander	Warmblood
29	22	w	Westphalian horse	Warmblood
30	8	m	Holsteiner	Warmblood
31	8	w	Mecklenburger	Warmblood
32	24	w	Mecklenburger	Warmblood
33	25	m	Mecklenburger	Warmblood
34	17	m	Special riding horse (Welsh B/Holsteiner)	Other
35	17	m	Hannoverian horse	Warmblood
36	8	m	Hannoverian horse	Warmblood
37	12	w	Tenesse Walking Horse/Holsteiner	Other
38	5	m	Trakehner	Warmblood
39	9	m	Andalusian horse	Other
40	11	w	Holsteiner	Warmblood
41	12	w	Hannoverian horse	Warmblood
42	8	m	Holsteiner	Warmblood
43	9	m	Holsteiner	Warmblood
44	8	m	Haflinger	Pony
45	8	m	German Riding Pony	Pony
46	20	w	Sachsen-Anhaltiner	Warmblood
47	2	w	Quarab	Other
48	11	m	Hannoverian horse	Warmblood
49	4	w	Quarab	Other

50	18	m	Friesian	Other
51	8	w	Holsteiner	Warmblood
52	21	w	Oldenburger	Warmblood
